# Innovative Applications and Challenges of Isothermal Amplification Technology in miRNA Detection

**DOI:** 10.2174/0113892029436961260112093518

**Published:** 2026-03-30

**Authors:** Xuebo Li, Zhonglin Zhang, Jian Zhu, Yi Zhang, Yongcan Guo

**Affiliations:** 1 Department of Laboratory Medicine, Clinical Laboratory Medicine Research Center, West China Hospital, Sichuan University, Sichuan Clinical Research Center for Laboratory Medicine, Chengdu, 610000, PR China;; 2 The 3RD Affiliated Hospital of Chengdu Medical College,Chengdu Pidu Distriet People's Hospital,Chengdu, 611730, PR China;; 3 Nanobiosensing and Microfluidic Point-of-Care Testing, Key Laboratory of Luzhou, Department of Clinical Laboratory, The Affiliated Traditional Chinese Medicine Hospital, Southwest Medical University, Luzhou, 646000, PR China

**Keywords:** CRISPR/Cas, isothermal amplification, miRNA, nanobiosensing, tumor biomarkers

## Abstract

A class of endogenous non-coding RNAs with a length of roughly 18–25 nucleotides is known as microRNAs (miRNAs). They have been established as the best biomarkers for early cancer diagnosis, molecular subtyping, and prognostic evaluation. They also play important roles in important biological processes like cell proliferation, differentiation, apoptosis, and tumorigenesis through post-transcriptional regulatory networks. Traditional detection technologies (such as quantitative Polymerase Chain Reaction (qPCR), Northern blot, and microarrays) exhibit high sensitivity, yet they rely on expensive thermocycling equipment, involve cumbersome procedures, and are time-consuming, making it difficult to meet the demands of clinical point-of-care testing. In recent years, isothermal amplification technologies, represented by loop-mediated isothermal amplification (LAMP), Rolling Circle Amplification (RCA), Strand Displacement Amplification (SDA), and Exponential Amplification Reaction (EXPAR), have emerged as a research frontier in the field of miRNA detection, by virtue of their advantages including elimination of thermal cycling requirements, fast reaction kinetics, high sensitivity, and simple operation. This study aims to systematically organize the core principles, latest advancements, and integrated innovation patterns of isothermal amplification-based miRNA detection technologies, and provide support for the development of miRNA detection platforms suitable for clinical point-of-care testing.

## 1 INTRODUCTION

A class of endogenous non-coding single-stranded RNAs with a length of roughly 18–25 nucleotides (nt) is known as microRNAs (miRNAs). They participate in important biological processes like cell proliferation, differentiation, apoptosis, and metabolic reprogramming by precisely regulating gene expression networks at the post-transcriptional level through sequence-specific complementary binding to the 3' Untranslated Regions (3'-UTRs) of target mRNAs [1-4]. Numerous studies conducted in recent years have shown that dysregulation of miRNA expression profiles is closely linked to the development, progression, and drug resistance of a number of human diseases, especially malignant tumors. As a result, miRNAs have emerged as extremely promising liquid biopsy biomarkers and precision medicine targets [3, 5]. Nonetheless, miRNAs have intrinsic properties such as a short sequence, low abundance, high sequence homology within the family, and vulnerability to RNase degradation in physiological fluids. Achieving highly sensitive, highly specific, quick, and convenient detection is extremely difficult due to these characteristics [6, 7].

High-throughput sequencing (NGS), microarray chips, Northern blotting, *in situ* Hybridization (ISH), and qPCR are currently the most widely used miRNA detection techniques [8, 9]. While NGS enables profiling of the entire transcriptome's miRNA repertoire in a single run, it has drawbacks such as expensive instruments, complex data analysis, and sensitivity limitations that depend on sequencing depth [10]. Despite their advantage of parallel detection, microarray chips suffer from a narrow dynamic range, significant batch-to-batch variations, and challenges in achieving absolute quantification [11, 12]. Even though northern blotting is regarded as one of the “gold standards” for its direct ability to separate mature miRNAs from their precursors, its application in high-throughput settings is limited due to laborious procedures, days-long turnaround time, and detection limits typically in the picomolar range [13, 14]. While probe permeability issues and background signal interference restrict the quantitative accuracy of ISH, this technique can visualize the spatial distribution of miRNAs within tissues [15]. As for qPCR, it has been widely used due to its high sensitivity; however, given the short sequence of miRNAs, it must rely on stem-loop or tailing reverse transcription strategies, which involve cumbersome procedures, require thermocyclers, and are prone to introducing biases. Additionally, in low-abundance samples, issues such as low amplification efficiency and insufficient specificity tend to arise [16-18]. Therefore, to address these current limitations, efforts should be made to develop new technological platforms capable of performing amplification and detection under isothermal conditions while eliminating the need for thermocyclers.

Due to its benefits, which include mild reaction conditions, low equipment requirements, short reaction times, high sensitivity, and strong specificity, Isothermal Amplification (IA) technology has become a popular alternative in the field of miRNA detection in recent years [19]. For the quick and accurate measurement of miRNAs, methods such as Rolling Circle Amplification (RCA), Strand Displacement Amplification (SDA), Exponential Amplification Reaction (EXPAR), Loop-Mediated Isothermal Amplification (LAMP), and enzyme-free catalytic amplification systems have been reported one after the other [20-22]. Nevertheless, non-specific amplification, primer-dimer interference, and complicated probe design are still issues with single isothermal amplification techniques. In order to overcome these obstacles, scientists have combined isothermal amplification with CRISPR/Cas systems, functionalized nanoprobes, and multi-signal amplification units to create portable, all-in-one, ultra-high-sensitivity biosensing platforms. This integration offers new methods for implementing miRNA in precision medicine and Point-of-Care Testing (POCT) [23, 24]. Against this backdrop, this study focuses on organizing the evolution of mainstream technical principles, key performance breakthroughs, and multi-technology integration mechanisms of isothermal amplification technology in miRNA detection, aiming to provide targeted theoretical references and practical guidance for optimizing the highly sensitive, specific, portable, and integrated miRNA detection technical schemes that meet the needs of point-of-care testing, as well as their clinical translation pathways.

## 2 TECHNIQUES FOR ISOTHERMAL AMPLIFICATION-BASED MIRNA DETECTION

### 2.1 Rolling Circle Amplification(RCA)

Due to its high sensitivity, high specificity, and “one-pot” reaction feature that does not require thermal cycling, Rolling Circle Amplification (RCA) technology has emerged as one of the most researched isothermal amplification techniques for miRNA detection [25]. Its fundamental idea entails two crucial steps, as shown in Fig. (**1**): To create a closed circular template, T4 DNA ligase first circularizes the linear padlock probe with the help of miRNA. Under isothermal conditions, Phi29 DNA polymerase then carries out rolling circle extension by utilizing its strand displacement activity and processivity. This achieves exponential signal amplification by producing long single-stranded DNA (ssDNA) products made up of hundreds to thousands of repeating units [26-28].

For endpoint detection, traditional RCA uses gel electrophoresis, which has a low throughput and low sensitivity. By combining various signal transduction platforms, such as fluorescence, colorimetry, electrochemistry, and functionalized nanomaterials, researchers have improved the detection performance and clinical applicability of RCA technology in recent years [29, 30]. Song *et al.* developed a method called Palindromic Hyperbranched Rolling Circle Amplification (PH-RCA), in which a dumbbell probe changes shape when miRNA is present to start RCA. Palindromic sequences then self-assemble to create a multibranched structure. This allows for accurate discrimination of single-base mismatches and attains a detection limit of 18.5 aM [31]. Self-Primed Extension (SPE) and RCA were combined by Shao *et al*. [32]. A strand-displacing polymerase continuously extends the signal after biotin-labeled RCA products are captured on a streptavidin-coated microplate surface. With this method, the detection limit is as low as 10 fM, and it shows good tolerance to the serum matrix. CRISPR/Cas signal amplification technology has been used by researchers to further increase the sensitivity of miRNA detection. Zhou *et al.* [33], for example, created an integrated system called T-ERCA that combines a CRISPR/Cas12a trans-cleavage system with dumbbell probe-triggered exponential RCA: By acting as an activator, the lengthy ssDNA product produced by RCA causes Cas12a to cleave fluorescent reporter probes in large quantities, facilitating secondary signal amplification. As shown in (Fig. **2**), this successfully profiles miRNA expression in various cell lines by lowering the detection limit to 0.31 fM.

Despite RCA's encouraging potential in miRNA detection, non-specific amplification and false positives caused by extremely similar sequences continue to be major obstacles to its clinical application [34, 35]. Research shows that lock probes can mis-circularize and produce increased background signals when sequence differences between members of the miRNA family are only 1–2 nucleotides [36]. In the future, a “Padlock Design-and-Operation Checklist” will be implemented to eliminate mis-circularization caused by homologous sequences: a 2-nt gap is positioned in the variable region and ligated with SplintR at 37R at 37on caused by homologous sequences: a 2-nt gapdT or C3-spacer is introduced to block off-target extension, reducing the background by≥5-fold; three single-base-mismatch synthetic targets must exhibit ligation efficiencies ≤ 5% (ΔCt ≥ 4.3), with PLC/NLC data disclosed in the main text; optional CRISPR/Cas13 secondary cleavage can further drive the false-positive rate to ≤ 1%. Coupling these measures with a microfluidic-RCA chip will provide an error-controlled and fully reproducible all-in-one solution for POCT and precision medicine.

Future developments could further lower false-positive rates and move RCA technology closer to Point-of-Care Testing (POCT) and precision medicine applications by improving the secondary structure of lock probes, introducing programmable nucleases (like CRISPR/Cas13) for real-time correction of non-specific products, and creating integrated microfluidic-RCA chips.

### 2.2 Loop-mediated Isothermal Amplification (LAMP)

First described by Notomi *et al.* [37]. in 2000, loop-mediated isothermal amplification (LAMP) is a “one-pot” method that can exponentially amplify nucleic acids at a fixed temperature (55–65°C). Targets are amplified by 109–1010 times in 15–60 minutes using Bst DNA polymerase with strand-displacing activity, which is characterized by the use of 4–6 specific primers (F3/B3, FIP/BIP, and optional loop primers LF/LB) that recognize 6–8 distinct regions of the target sequence [38-40]. Its canonical reaction pathway is as follows, as shown in (Fig. **3**): FIP anneals to the template and extends to form a stem-loop structure; the F3 primer then initiates strand displacement; multiple cycling produces dumbbell-shaped intermediates with terminal stem-loops; and finally, a large number of “cauliflower-like” long-stranded DNA products with repeating target sequences are produced.

However, because miRNA is only about 22 nt long, it is challenging to directly meet the 100–200 bp targets that LAMP typically requires [41]. Two approaches have been suggested by researchers to deal with this: (1) Stem-loop primer adaptation: Park Jee *et al.* [42] created chimeric primers that allow miRNA to directly initiate LAMP as a “trigger strand” without reverse transcription. The 5' end of these primers is complementary to miRNA, while the 3' end contains LAMP inner primer sequences. Similarly, the target miRNA was used by Ye *et al.* [43] to self-fold and extend with DNA in order to prime LAMP reactions. (2) Dumbbell template bridging: In a miRNA-LAMP system (Fig. **4**), SplintR ligase joins miRNA-mediated Linker A/B probes into dumbbell-shaped templates, which are then amplified by Bst 2.0 polymerase. This technique was developed by Wu *et al.* [44]. This achieves seven-order linear quantification within 100 fM–10 nM, with a detection limit as low as 100 fM, when used in conjunction with EvaGreen real-time fluorescence monitoring.

LAMP is being combined with several cutting-edge technologies to create multimodal cooperative amplification strategies in an effort to further reduce non-specific amplification and improve sensitivity [45, 46]: (1) RCA-LAMP cascade: Cao *et al.* [47] achieved a detection limit of 500 aM by using RCA-generated long single-stranded products as LAMP templates. The complexity of the LAMP primer is greatly decreased by this RCA pre-amplification.(2) LAMP-CRISPR/Cas12a cascade: Following LAMP amplification, Mao's group [48] added Cas12a using fluorescent reporter probes. By using Cas12a's trans-cleavage activity to amplify secondary signals, they were able to achieve single-base resolution and a detection limit of 0.1 fM.

In conclusion, even though LAMP offers a lot of benefits for point-of-care miRNA detection, the following issues still need to be fixed: (1) Non-specific amplification and primer dimerization: Erroneous amplification is easily triggered by minor sequence variations within miRNA homologous families. (2) Multiplex detection limitations: The implementation of multiplex LAMP is limited by crosstalk between complex primer sets and multicolor fluorescent probes. (3) Bottlenecks in field deployment: Because conventional LAMP still uses benchtop isothermal instruments, it must be further integrated with portable fluorescence readers, paper-based devices, or microfluidic chips. Future studies should concentrate on the following areas: (1) high-specificity primer design guided by machine learning, (2) improved mismatch discrimination *via* artificial base pairs or locked nucleic acid (LNA) modifications, (3) Synchronous multi-miRNA detection made possible by dual-compartment microfluidic-LAMP chips, and (4) Low-cost fluorescence/colorimetric readout systems that work with smartphones to enhance LAMP clinical translation in environments with limited resources.

### 2.3 Strand Displacement Amplification (SDA)

Strand-displacing DNA polymerases and restriction endonucleases (also known as nicking enzymes) work in concert to achieve exponential target amplification in Strand Displacement Amplification (SDA), an isothermal nucleic acid amplification technique that can be used at 55–65°C [49, 50]. Target miRNA anneals to a hairpin template, the enzyme nicks specific sites, polymerase facilitates extension and downstream strand displacement, and the single-stranded DNA template regenerates cyclically. This is its basic workflow. In just a few minutes, this cascade produces a large number of double-stranded products [51, 52]. Currently, the main miRNA detection strategies based on SDA include (Note: The limit of detection (LOD) data, disease discrimination cases, and clinical application explorations of the following strategies are preliminary research results):(1) A “one-pot” exponential SDA (E-SDA) system was created by Shi *et al.* [53]. in which two rounds of circular strand-displacement cycles (polymerization-cleavage-displacement, Fig. **5**) are initiated by target miRNA. It greatly outperforms traditional linear SDA, achieving signal amplification in 90 minutes with a detection limit of 16 zmol without the need for reverse transcription. (2) CRISPR-coupled SDA: Using miRNA as an SDA initiator, Chi *et al.* [54] developed a CRISPR-Cas14a-coordinated approach (Fig. **6**). G-rich single-stranded DNA is produced by target miRNA driving SDA, which triggers Cas14a's trans-cleavage activity for secondary signal amplification. In 30 μL serum samples, this technique successfully distinguishes cholangiocarcinoma patients from healthy controls, achieves an ultralow detection limit of 0.68 aM, and accurately separates miRNA-21 from single-base mutants, exhibiting remarkable clinical utility. (3) Micro-nano integrated SDA: Researchers have combined SDA with RCA cascades, magnetic nanoparticles (MNPs), or microchips to further increase sensitivity and throughput. For example, Xie *et al.* [55] enabled high-throughput parallel analysis by combining microchip electrophoresis with SDA and achieving a detection limit of 0.02 fM for miRNA-21 in a 5 μL reaction volume. The MNPs-SDA electrochemical sensor was created by Xu *et al.* [56] and successfully applied to the sera of breast cancer patients. MNP-conjugated capture probes enrich target miRNA in complex samples, achieving a sensitivity of 0.5 fmol·L^−1^. Zhou and colleagues [57] designed an RCA-SDA cascade: By producing long single-stranded templates for exponential SDA amplification, RCA pre-amplification enables femtomolar-level multiplex detection of miRNAs linked to colorectal cancer with noticeably higher specificity. Additionally, creating target-specific primers and probes is essential to raising the precision of miRNA detection. High-fidelity primers and probes significantly improve target miRNA selective recognition in SDA systems while lowering non-specific amplification [58, 59]. According to Li's research, better primer design increases specific binding to target miRNAs, leading to increased sensitivity and specificity [60].

The following bottlenecks still exist despite SDA's benefits, which include minimal reaction systems and thermal cycling independence:(1) strand-displacement byproduct accumulation that increases background signals; (2) reduced nicking enzyme activity when targeting complex secondary structures or miRNAs with high GC content; and (3) instability in detection as a result of serum RNase-mediated miRNA degradation. Future developments could concentrate on: (1) improving mismatch discrimination and probe stability through Locked Nucleic Acid (LNA) or 2'-O-methyl modifications, (2) creating integrated microfluidic-SDA chips that would allow for completely automated sample-to-answer workflows, and (3) constructing Cas14a-SDA dual-target logic gates to enable multiplex miRNAs to be precisely discriminated at the same time. With these tactics, SDA is well-positioned to advance from bench to bedside as a reliable tool for miRNA detection in liquid biopsy for cancer.

### 2.4 Enzyme-free Amplification Technologies

Enzyme dependency is totally eliminated by enzyme-free amplification technologies, which use entropy-driven strand displacement for signal amplification and programmable self-assembly of DNA molecules. These methods are important innovations for ultrasensitive miRNA detection because they combine benefits like low cost, high stability, and simplicity of automation [61-65]. Catalytic Hairpin Assembly (CHA), Hybridization Chain Reaction (HCR), and Entropy-Driven Amplification (EDA) are examples of contemporary mainstream techniques. By suppressing background noise and greatly expanding the linear detection range, these methods can be used singly or in combination.

#### 2.4.1 Catalytic Hairpin Assembly (CHA)

The target miRNA is used in Catalytic Hairpin Assembly (CHA) to cause two metastable hairpin probes to cascade hybridize. To achieve “target-catalyzed” signal amplification, a fresh round of conformational switching is catalyzed by each target binding event [66, 67]. Dong *et al.* [68] used flow cytometry to detect Fluorescence Resonance Energy Transfer (FRET) signals after anchoring CHA reactions on magnetic bead surfaces. The detection of miRNA-155 at 2 pM was made possible by the significant reduction of fluorescence background caused by the micrometer-scale spatial confinement of beads (Fig. **7**). Research shows that CHA can detect miRNA at picomolar concentrations with high sensitivity [69], which is frequently impossible with traditional techniques. To further improve detection sensitivity and specificity, CHA can be combined with fluorescence imaging or electrochemical sensors [70].

#### 2.4.2 Hybridization Chain Reaction (HCR)

With the help of miRNA-induced alternating polymerization of two DNA hairpins (H1/H2), Hybridization Chain Reaction (HCR) produces double-stranded DNA nanowires that are n times longer and accomplish geometric signal amplification. Researchers have investigated new approaches such as localized HCR, migrating HCR, and multibranched HCR to improve HCR efficiency [71-73], which has greatly increased detection throughput and sensitivity [74].

Among the notable implementations are the HCR-driven fiber-embedded microfluidic chip (HCR-FMB) built by Lu *et al.* [75]. It detects serum let-7a in 6 minutes with a detection limit of 100 pM and remarkable specificity by combining FRET and evanescent wave fluorescence. Tetrahedral DNA framework (TDN)-assisted CHA-HCR dual amplification was used by Zhang *et al.* [76] to create a cascade amplification system (Fig. **8**). When used for *in situ* imaging of let-7a in living cells, the TDN nanostructure spatially colocalizes CHA and HCR reactions, shortening reaction pathways to reach an ultralow detection limit of 8.51 pM.

#### 2.4.3 Entropy-Driven Amplification (EDA)

Entropy-Driven Amplification (EDA) uses strand displacement reactions powered by “entropy gain and free energy reduction” to detect miRNAs in an enzyme-free, highly specific manner [77, 78]. The following are important EDA-based miRNA detection techniques: (1) Surface-enhanced EDA: Cai *et al.* [79] designed two EDA reaction layers on the surface of gold nanoparticles. When combined with fluorescence quenching/recovery mechanisms, this allows for intracellular imaging of miRNA-21 that can differentiate between healthy and cancerous cells; and (2) CHA-EDA cascade: The entropy-driven reaction was started by Huang *et al.* [80] using CHA products as fuel strands (Fig. **9**). The sensitivity of miR-208a detection is increased to 1.3 fM by this two-stage amplification in a single tube, which is a 100-fold improvement over traditional CHA.

Despite the impressive performance of enzyme-free amplification technologies, they still face bottlenecks such as increased background signals caused by the adsorption of hairpin probes by non-target nucleic acids in complex samples, reduced single-base discrimination due to hairpin cross-reactivity, and highly temperature- and salt concentration-dependent kinetics of EDA reactions, resulting in suboptimal field stability that requires improvement. Future strategies may involve introducing locked nucleic acid (LNA) or peptide nucleic acid (PNA) modifications to enhance probe specificity, developing AI-assisted hairpin design platforms, and integrating with microfluidic platforms. These approaches will help overcome the barriers in complex sample analysis and clinical scenarios, accelerating the translation of miRNA biomarkers into point-of-care diagnostics and precision medicine.

### 2.5 Exponential Amplification Reaction (EXPAR)

Short DNA templates, nicking enzymes, and strand-displacing DNA polymerases are used in the Exponential Amplification Reaction (EXPAR) to accomplish exponential amplification at isothermal temperatures (55–60°C) [81, 82]. Its template has an Nt—BstNBI nicking site along with two repeating sequences. A complementary strand is produced by polymerase extension when miRNA hybridizes with the template's 3' end. Following this strand's cleavage by the nicking enzyme, new primers are created, starting additional extension and downstream strand displacement. Target miRNA is amplified exponentially by this “extension-cleavage-displacement” cycle (Fig. **10**) [83]. EXPAR has become a potent tool for point-of-care miRNA detection due to its reverse transcription-free operation, ultrahigh sensitivity (zmol level), and quick kinetics (<30 min).

EXPAR performs exceptionally well in miRNA detection, especially in terms of reaction efficiency and sensitivity [84-86]. Studies have demonstrated that portable detection platforms integrated with EXPAR exhibit application potential in early-stage feasibility studies. They enable the detection of multiple miRNAs in urine samples within 30 minutes, with an accuracy of 83% [87]. Pan *et al.* [88] used thermodynamic template optimization to achieve a 90% reduction in background amplification and a detection limit of 10 zmol for let-7a. By using hairpin templates to block non-specific interactions, Peng *et al.* [89] created a self-priming EXPAR (SPEXPAR)-lateral flow colorimetric platform that allows for quantitative miRNA-155 detection through gold nanoparticle (AuNP) color transitions in 35 minutes over a linear range of 0.41 pM–100 nM.

Despite the many advantages of EXPAR technology, background amplification signals remain a major challenge in EXPAR reactions [90]. The main sources of non-specific background amplification signals are: (1) primer-mediated non-specific amplification, (2) template-induced non-specific amplification, and (3) DNA polymerase-mediated non-specific amplification. The main causes of EXPAR background signals and improved measures to suppress background amplification signals will be discussed. The high non-specific background of EXPAR stems from primer mismatches, template self-hybridization, and non-specific extension by polymerases. The following text reviews non-specific suppression strategies from three aspects: molecular design, chemical modification, and system engineering.

#### 2.5.1 Primer Optimization

Due to their short sequence length and high GC content, miRNAs are more likely to exhibit partial complementarity with non-target sequences, which can result in mismatch extension and negatively impact the sensitivity and reliability of detection [91]. Moreover, non-specific binding greatly increases background signals when primers have sequence similarities with off-target regions [92]. Non-specific binding can be significantly decreased by using machine learning for secondary structure prediction, optimizing the design of miRNA primers (GC content, length, and target match specificity), or adding LNA/2′-OMe modifications to improve ΔTm.

#### 2.5.2 Template Engineering

One of the main sources of EXPAR background signals is non-specific template amplification, which results from transitory hybridization at template 3'-ends [93, 94]. When the 3'-termini of two templates come into contact during nucleic acid amplification, complementary base pairing may result in the formation of unstable duplex structures. Furthermore, templates have the ability to self-fold into hairpin patterns, which results in the temporary production of duplexes at their 3'-termini. By activating DNA polymerase, these structures behave as pseudo-priming sites, starting amplification without the need for valid initiator strands. Fig. (**11**) [95] shows the four main unintentional amplification paths of autonomous templates. Importantly, polymerase extension can be primed by complementary sequences at 3'-ends without the need for particular primers, increasing background noise and lowering detection accuracy [96].

In order to decrease EXPAR background signals, structural modification techniques that prevent the non-specific amplification of EXPAR templates primarily consist of: (1) Template end-capping technology: this technique stops non-specific amplification by altering the nucleic acid template's 3' end to stop polymerase extension. Research has demonstrated that closing the 3' end with inverted T, C3 Spacer, PNA, and other modifications can not only successfully stop polymerase extension but also improve template stability, increasing the detection's sensitivity and specificity [82, 97, 98]. Furthermore, a study conducted by Chen *et al.* [99] showed that modifying the template's 5' end with biotin can lower its melting temperature (Tm), which enables miRNA to preferentially bind to the 3' end and extend, minimizing template loss and increasing detection efficiency. (2) Secondary Structure Design: Nonspecific signals can be efficiently suppressed by the secondary structure design of the template, particularly the hairpin structure. Using the hairpin conformation to prevent template self-extension, Guo *et al.* [100] created an exp-Hairpin template with embedded cut sites in the stem region. This resulted in a theoretical amplification efficiency of >2^N^ and a detection limit of miRNA-21 at 5.84 fM, greatly increasing the reaction's specificity (Fig. **12**). (3) Chemical modification techniques: Thioester bonds, methylation, phosphorylation, and glutaraldehyde treatment can all improve the sensitivity and accuracy of detection by increasing template rigidity and decreasing non-specific binding [101-103].

#### 2.5.3 System Engineering

In order to limit the template's nonspecific amplification, the system engineering approach primarily entails adding additional substances to the reaction system. Primarily: (1) Additive strategy: single-stranded binding proteins (SSB) can reduce the background by three orders of magnitude; 5–10% glycerol or DMSO can reduce π-π stacking between templates [95, 104]. (2) Nanomaterial shielding: By using π-π interactions to adsorb free templates, Graphene Oxide (GO) stops polymerase from binding non-specifically, enabling detection at the 5 aM level with a sensitivity that is 10^4^ times higher than that of conventional EXPAR [105]. (3) 1,6-hexanediol and two-dimensional CoOOH nanosheets can prevent template self-assembly, which reduces background signal by more than 95% [106].

#### 2.5.4 DNA Polymerase-mediated Non-specific Amplification

Random primer formation and extension are crucial steps in DNA polymerase's de novo synthesis capability, which is based on structural characteristics and catalytic mechanisms and is the main source of non-specific amplification [107, 108]. The ability of DNA polymerases to displace existing DNA strands during nascent strand synthesis, known as strand-displacing activity, is the primary driving force behind the synthesis of random primers in the absence of target sequences.

As an illustration, the enzyme Bst Exo-polymerase has a high affinity for binding purine-rich DNA motifs. In particular, by promoting polymerase-primer interactions, dG at the 3'-terminus of primers encourages random primer formation [109]. Random primers are extended to create new DNA strands after polymerase binding. Any section of the template DNA could act as a primer extension initiation site during this procedure, resulting in non-specific amplification [110, 111]. We advise using modified nucleotides with greater fidelity and optimizing primer design to steer clear of dA/dG-rich 3'-termini in order to reduce random primer generation. These methods greatly improve the reliability and specificity of amplification.

In summary, the EXPAR technology shows great potential in the field of nucleic acid detection, but its non-specific amplification remains a critical bottleneck to be addressed. Based on the aforementioned strategies, a preliminary background suppression protocol can be established: using 3'-end blocked hairpin templates (containing a nicking site) in combination with Nt.BstNBI nuclease and Bst Exo^−^ polymerase, with the reaction system supplemented with 5% glycerol and single-stranded binding protein (SSB) to reduce non-specific interactions. It specifies that the fluorescence rise time of the No-Template Control (NTC) should be ≥60 minutes (delayed by ≥30 minutes compared to the target), with a background slope ≤0.02 RFU/min (within 20-60 minutes). For crude matrices such as serum and urine, blinded validation is required, specifying processing details (*e.g*., serum inactivation at 56°C + centrifugation), blinded detection accuracy (≥85% for serum, ≥80% for urine), and concentrations and impacts of interferents (*e.g*., hemoglobin), thus providing replicable standards for the stable application of EXPAR.

Future development of EXPAR will focus on: (1) AI-based template-primer co-design platforms to achieve single-base resolution, (2) integration of microfluidics with EXPAR chips to enable sample-in-result-out POCT modes, and (3) multiplex target EXPAR encoding systems to meet clinical needs for simultaneous quantification of multiple miRNAs. With the maturation of background suppression strategies and device miniaturization, EXPAR is expected to achieve widespread translation in the fields of early tumor screening, prognostic monitoring, and home self-testing.

## 3 DISCUSSION

### 3.1 Core Value of Isothermal Amplification in miRNA Detection

Isothermal amplification technologies, with core advantages of “no need for thermal cycling, rapid reaction, and high sensitivity,” have significantly broken through the technical bottlenecks of conventional qPCR in miRNA detection, providing a new methodological basis for early cancer screening, companion diagnosis, and prognostic monitoring. This review systematically summarizes the principal innovations, performance limits, and clinical validation of strategies such as RCA, LAMP, SDA, EXPAR, and enzyme-free amplification (CHA/HCR/EDA) in miRNA quantification (Table **1**). Their common advantages are reflected in: (1) Simple operation, achieving detection limits at the aM–zmol level with a single-temperature reaction, (2) Seamless coupling with multidisciplinary technologies (*e.g*., CRISPR, nanomaterials, microfluidics) to realize secondary signal amplification and on-site detection, and (3) Good compatibility with short, low-abundance, and highly homologous miRNAs.

### 3.2 Limitations of Current Technologies

Despite the significant advantages of isothermal amplification technologies in miRNA detection, several challenges remain: (1) Non-specific amplification and cross-reactivity have not been fully eradicated, resulting in prominent background noise in the low-copy number range, (2) In multiplex parallel detection of targets, complex interactions between primers and templates reduce quantitative accuracy, (3) RNases, inhibitors, and pH fluctuations in complex biological matrices (*e.g*., whole blood, tissue lysates) easily interfere with amplification efficiency, and clinical reproducibility requires further verification, and (4) Most existing platforms remain in the stage of laboratory proof-of-concept, lacking complete solutions for deep integration with commercial POCT devices.

Regarding the core issues directly related to the standardization of sample processing and data reliability among the aforementioned challenges (such as interference from complex matrices, insufficient reproducibility caused by variations in sample pretreatment, and inconsistent quantitative calibration methods), a series of Standardized Operating Procedures (SOPs) adapted to isothermal amplification technologies has been initially developed based on existing studies. These SOPs provide practical and implementable solutions for enhancing the robustness of detection. Tables (**2** and **3**) below systematically sort out key operational points and technical adaptability from the perspectives of pre-analytical variable control and data standardization, respectively, offering references for experimental design and quality control in clinical translation.

### 3.3 Future Perspectives

In the future, leapfrog improvements in isothermal amplification miRNA detection can be made from the following dimensions: (1) Molecular design driven by algorithms: A “sequence-structure-performance” prediction model is developed using AI-assisted thermodynamic calculations and machine learning to achieve the automatic and ideal design of primers, templates, and probes, greatly reducing the experimental iteration cycle, (2) The multiplexing strategy at the system level: The requirements of tumor molecular subtyping and drug resistance monitoring are met by combining isothermal amplification with a high-throughput decoding platform to achieve absolute quantification of >5 miRNAs in a single reaction using programmable barcodes, microarray partitioning, or digital microdroplet technology. For example, an isothermal LAMP system employing 55-µm droplets (λ = 0.06) was used to compartmentalize individual templates; the predicted droplet collision rate was ≤ 0.55%. A 12-dimensional code space generated by four orthogonal fluorophores × three intensity levels was narrowed to six dimensions that cover liver-cancer-related miRNAs. After AI-based primer filtering, the decoding error remained < 1% and serum validation yielded r = 0.93 *versus* qPCR, establishing a reproducible, >5-plex, isothermal multiplexing paradigm, (3) Anti-interference system of clinical quality: To guarantee the robustness of detection in complex samples, an integrated sample pre-processing module (rapid lysis, inhibitor adsorption, and RNA enrichment) was created. Additionally, internal reference correction and real-time quality control probes were added, and (4) End-to-end automated platform: It deeply integrates isothermal amplification (with precise temperature control and minimal fluctuations), smartphone-based optical reading (equipped with a custom dark box and specialized optical filter, achieving a significantly enhanced optical signal-to-noise ratio), cloud-based AI interpretation (with high accuracy), and blockchain-based data management (with an extremely low risk of tampering). Through fluorescent microsphere calibration targets and an auto-exposure locking mechanism, it effectively controls device variability, thereby constructing a full-link closed-loop system encompassing “sample-result-report.” This platform features a short total processing time and greatly simplified operational procedures, and is truly applicable in primary hospitals, emergency vehicles, and home self-testing scenarios.

## CONCLUSION

In conclusion, the transition from “methodological innovation” to “clinical transformation” in isothermal amplification technology has reached a critical window. In the future, it is anticipated that collaborative innovation in algorithms, materials, chips, and data science will advance miRNA detection from the lab to the final mile of public health management.

## Figures and Tables

**Fig. (1):**
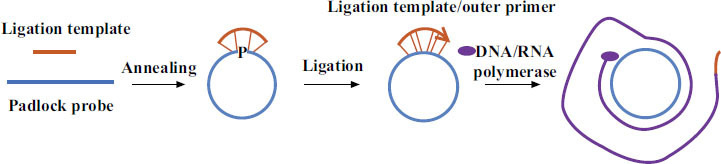
Schematic diagram illustrating the principle of RCA for miRNA detection.

**Fig. (2):**
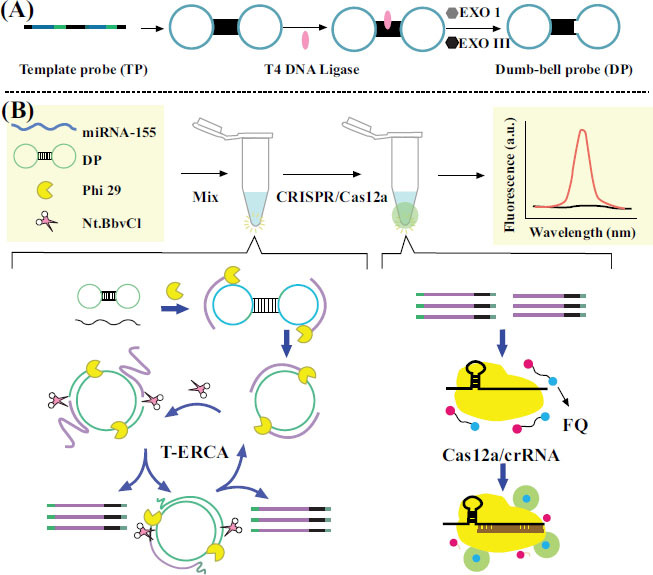
**(A, B).** T-ERCA combined with CRISPR/Cas12 system: detection schematic.

**Fig. (3):**
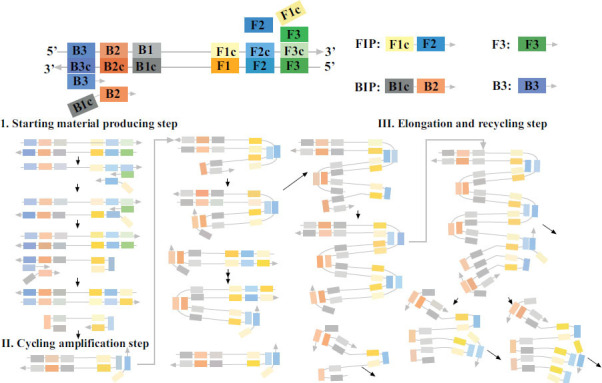
Schematic illustration of the LAMP working principle.

**Fig. (4):**
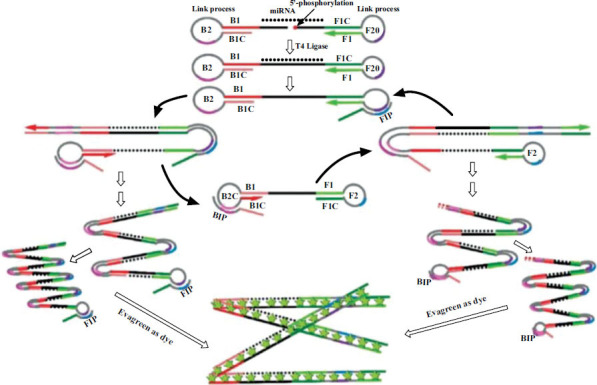
Schematic illustration of miRNA detection using the miRNA-LAMP system. Reproduced from Wu *et al*., *Int. J. Anal. Chem.*, 2023, under the terms of the Creative Commons Attribution 4.0 International License (CC BY 4.0) [44].

**Fig. (5):**
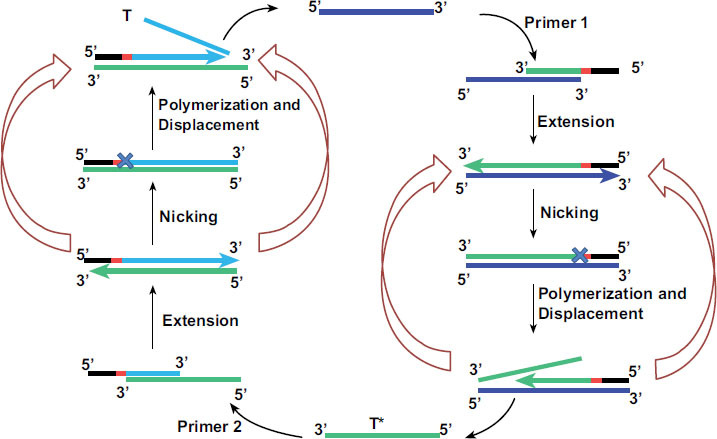
Schematic illustration of miRNA-triggered Exponential SDA (E-SDA).

**Fig. (6):**
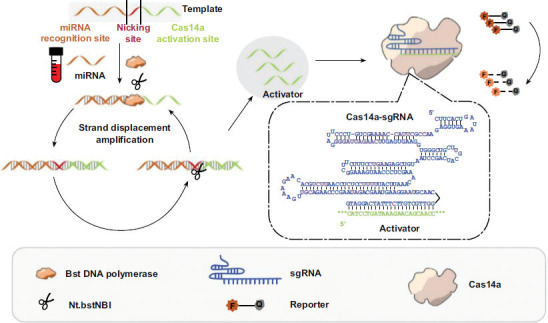
Schematic illustration of miRNA detection mediated by the Cas14-SDA system.

**Fig. (7):**
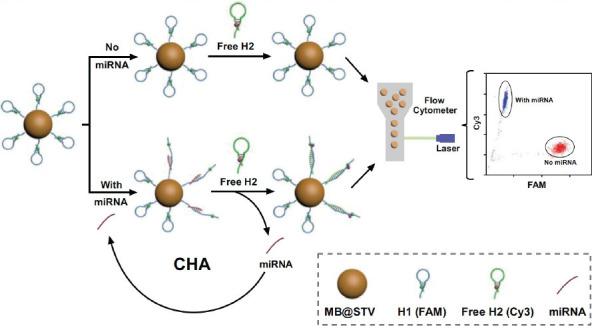
Schematic illustration of the miRNA detection strategy using CHA-coupled flow cytometry (FRET).

**Fig. (8):**
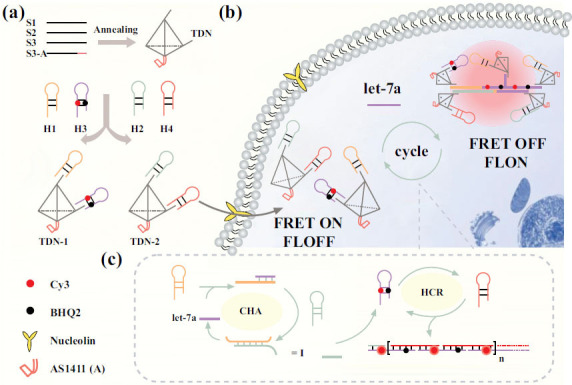
(**a-c**). Schematic illustration of the working principle for detecting let-7a using the TDN-CHA-HCR system.

**Fig. (9):**
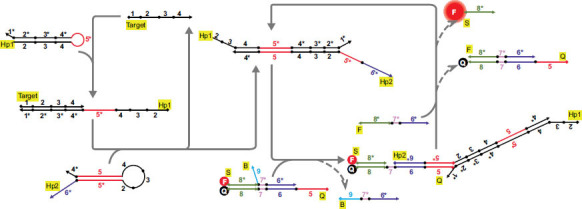
Schematic illustration of the working principle for the enzyme-free cascade amplification biosensor detecting microRNA using the EDA-CHA system.

**Fig. (10):**
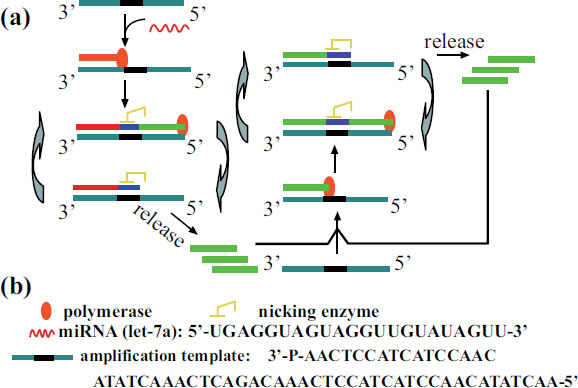
(a and b). Schematic illustration of microRNA detection using EXPAR.

**Fig. (11):**
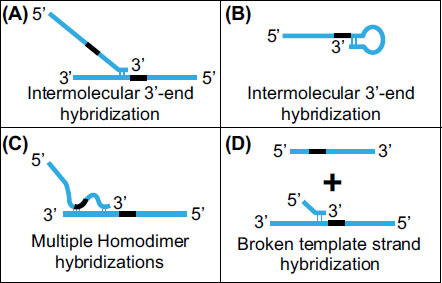
(A) Inter-template 3'-end hybridization. (B) Intra-template 3'-terminal hairpin formation. (C) Transient template-template interactions. (D) Hybridization between template fragments and intact templates.

**Fig. (12):**
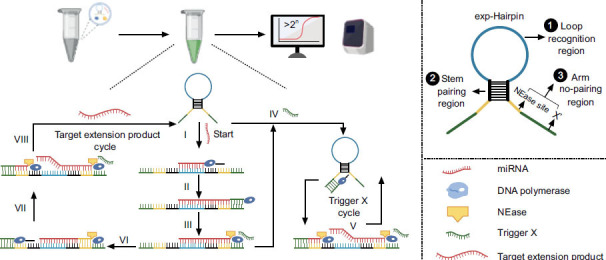
Schematic illustration of the working principle for miRNA detection using the exp-Hairpin strategy.

**Table 1:** Characteristics of different isothermal amplification methods.

**Method**	**Type**	**Target**	**Reaction Volume**	**Detection Time**	**Detection** **Range**	**LOD**	**References**
RCA	CRISPR-Cas12a	miR-21	110 μL	70 min	100 pM-10 nM	8.1 pM	[27]
	DNA(HD)-RCA	miR-21	30 μL	90 min	1 aM-1 pM	39.7 aM	[28]
	PH-RCA	miR-141	N/A	30 min	100 aM-1 nM	18.5 aM	[31]
	T-ERCA	miR-155	20 μL	100 min	1 fM-5 nM	0.31 fM	[33]
LAMP	Ligation-LAMP	miR-155	50 μL	110 min	10 fM-100 pM	15 fM	[39]
	RT-THSP-LAMP	miR-152	20 μL	120 min	10 fM-3 nM	10 fM	[43]
	miRNA-LAMP	miR-200a	30 μL	180 min	100 fM-5 nM	100 fM	[44]
	CAL-LAMP	let-7a	200 μL	73 min	100 aM-100 fM	0.1 fM	[48]
SDA	B-HCR	miR-21	20 μL	150 min	0-60 nM	11.3 pM	[49]
	SDA	let-7a	10 μL	90 min	0.1 zmol-1.0 pmol	16 zmol	[53]
	Cas14SDA	miR-21	30 μL	60 min	0.68-50 pM	680 fM	[54]
	CRISPR/Cas12a	miR-21	20 μL	150 min	0.5 fM-100 pM	0.5 fM	[56]
CHA	split-G4-CHA	miR-21	20 μL	70 min	10 fM-100 nM	37 fM	[66]
	FCM-CHA	let-7a	10 μL	180 min	0.7 pM-300 pM	0.7 pM	[68]
	FA-CHA	miR-21	N/A	240 min	1.0-19.0 nM	52.0 pM	[67]
	ECL-CHA	miR-21	5 μL	120 min	1 aM-1 nM	0.02 aM	[70]
HCR	CRISPR/Cas12a	miR-21	N/A	60 min	250 fM-10,000 pM	17.6 amol	[72]
	HCR-FMB	let-7a	20 μL	6 min	3.0-1000 nM	100 pM	[75]
	TDN-CHA-HCR	let-7a	N/A	60 min	0.05-5 nM	8.51 pM	[76]
EDA	CRISPR/Cas13a	miR-17	20 μL	45 min	1 fM-100 pM	1.05 fM	[77]
	AuNPs-EDA	miR-21	100 μL	120 min	5 pM-25 nM	5 pM	[79]
	CHA-EDA	miR-21	N/A	150 min	50 fM-1 pM	1.3 fM	[80]
EXPAR	EXPAR	let-7a	10 μL	30 min	0.1 zmol-1.0 pmol	0.1 zmol	[83]
	SPEXPAR	miR-155	10 μL	40 min	10 fM-1 μM	0.41 pM	[89]
	exp-Hairpin EXPAR	let-7a	30 μL	34 min	10 fM-10 nM	5.84 fM	[100]
	GO/SSB	trigger	20 μL	60 min	5 aM-50 fM	5 aM	[105]
	CoOOH	miR-122	20 μL	N/A	50 aM-500 fM	151 ymol	[106]

**Table 2:** Operating specifications for pre-analytical variable control.

**Module**	**Key Operations**	**Critical Control Points**	**Compatible Isothermal** **Technologies**	**Limitations**	**Application** **Scenarios**	**References**
Pre-analytical Variable Control	1. Sample collection and anticoagulation	- Blood/serum: EDTA tubes; centrifuge 3000×g, 4°C, 10 min (≤30 min post-collection). Avoid heparin (47% enzyme inhibition). - Urine/CSF: RNase-free tubes + 0.1% DEPC.	- LAMP (heparin-tolerant); - RCA (strict anticoagulant control).	- EDTA reduces miR-451a recovery (~8%); - Heparin impairs SDA (23% efficiency loss).	- Clinical blood/fluid testing; - Multicenter studies.	[112, 113]
	2. Storage and freeze-thaw control	- Isolate *via* ultracentrifugation (100,000×g, 4°C, 70 min) or ExoQuick. - Lyse with 0.5% Triton X-100 (release rate: 62%→91%).	- EXPAR (degradation-sensitive); - LAMP (mild tolerance).	- miR-16 declines 12–15% after 6-month -80°C storage ; - Prolonged ice thawing ↑RNase (2.3×).	- Biobank samples; - Low-abundance miRNA detection.	[114]
	3. miRNA extraction and lysis	- Isolate *via* ultracentrifugation (100,000×g, 4°C, 70 min) or ExoQuick. - Lyse with 0.5% Triton X-100 (release rate: 62%→91%).	- RCA (requires efficient lysis); - SDA (reliant on free miRNAs).	- Ultracentrifugation loses ~30% exosomes (*vs*. 15% with kits); - Triton >0.5% ↓LAMP (18%).	- Liquid biopsies (tumor markers); - Extracellular vesicle research.	[115]
	4. Matrix compatibility and inhibition checks	- Complex matrices: 1:10 dilution or inhibitor columns. - Simple matrices: 1× TE (pH 8.0) + 0.1% SDS. - Inhibitor test: ΔCt <2 for miR-16.	- LAMP (anti-interference); - EXPAR (requires ≥1:50 dilution).	- Complex matrices (whole blood, feces); - Novel matrices (saliva).	- Complex matrices (whole blood, feces); - Novel matrices (saliva).	[116]

**Table 3:** Operating specifications for standardization strategies.

**Module**	**Key Operations**	**Critical Control Points**	**Compatible Isothermal Technologies**	**Limitations**	**Application Scenarios**	**References**
Normalization Strategies	1. Exogenous spike-in calibration	Use non-human miRNAs (*e.g*., cel-miR-39) at 50 fM (added post-collection). Require ≥70% recovery.	- All (match target efficiency); - Prefer LAMP/SDA (broad linear range).	- Multicenter studies; - Assay validation.	- Multicenter studies; - Assay validation.	[117]
	2. Reference miRNA selection	Candidates: miR-16/miR-451a (serum); miR-122/miR-93 (tissues). Select *via* geNorm (M-value <0.5).	Single-target detection; avoid miRNA families (cross-amplification).	- Clinical typing; - Tissue expression analysis.	- Clinical typing; - Tissue expression analysis.	[118]
	3. Global mean normalization (multiplex)	For ≥5 miRNAs: calculate mean Ct; exclude outliers (±3 SD). Normalization: target Ct – mean Ct.	Multiplex digital LAMP; chip-based amplification (≥10 miRNAs).	- High-throughput screening; - Novel samples.	- High-throughput screening; - Novel samples.	[119]
	4. System quality control	RNase control: DEPC consumables + 0.1 U/μL RNasin. - NTC signal <10% of target; CV <15% (3 replicates).	All (EXPAR/RCA: contamination-sensitive); automated platforms.	- Clinical lab QC; - Automated POCT.	- Clinical lab QC; - Automated POCT.	[120, 121]

## LIST OF ABBREVIATIONS

EXPAR: Exponential Amplification Reaction
IA: Isothermal Amplification
LAMP: Loop-Mediated Isothermal Amplification
POCT: Point-Of-Care Testing
qPCR: quantitative Polymerase Chain Reaction
RCA: Rolling Circle Amplification
SDA: Strand Displacement Amplification

## References

[1] Ye J., Xu M., Tian X., Cai S., Zeng S. (2019). Research advances in the detection of miRNA.. J. Pharm. Anal..

[2] Lee C. (2022). A combinational approach for more efficient miRNA biosensing.. Curr. Genomics.

[3] Al-Hawary S.I.S., Saleh R.O., Mansouri S., Noraldeen S.A.M., Alawadi A.H., Kareem A.H., Alsalamy A., Shakir M.N., Alasheqi M.Q., Mustafa Y.F. (2024). Isothermal amplification methods in cancer-related miRNA detection; A new paradigm in study of cancer pathology.. Pathol. Res. Pract..

[4] Hu X., Yin G., Zhang Y., Zhu L., Huang H., Lv K. (2023). Recent advances in the functional explorations of nuclear microRNAs.. Front. Immunol..

[5] Yao Y., Chao M., Yin F., Wang Q., Sun H., Wang Z., Xiang Y. (2025). A three-way junction-mediated SDA cascade CHA system for miRNA detection.. Talanta.

[6] Xia J., Liu Z., Gao S., Wang Q., Xu J., Wu H. (2022). Intermolecular and intramolecular priming co-directed synergistic multi-strand displacement amplification empowers ultrasensitive determination of microRNAs.. Anal. Chem..

[7] Qu X., Ren X., Su N., Sun X., Deng S., Lu W., Huang Q. (2023). Isothermal exponential amplification reactions triggered by circular templates (cEXPAR) targeting miRNA.. Mol. Biol. Rep..

[8] Chalasani N.P., Porter K., Bhattacharya A., Book A.J., Neis B.M., Xiong K.M., Ramasubramanian T.S., Edwards D.K. 5th, Chen I., Johnson S., Roberts L.R., Kisiel J.B., Reddy K.R., Singal A.G., Olson M.C., Bruinsma J.J. (2022). Validation of a novel multitarget blood test shows high sensitivity to detect early stage hepatocellular carcinoma.. Clin. Gastroenterol. Hepatol..

[9] Matulić M., Gršković P., Petrović A., Begić V., Harabajsa S., Korać P. (2022). miRNA in molecular diagnostics.. Bioengineering.

[10] Liu K., Tong H., Li T., Wang X., Chen Y. (2020). Research progress in molecular biology related quantitative methods of MicroRNA.. Am. J. Transl. Res..

[11] Juanes-Velasco P., Arias-Hidalgo C., Landeira-Viñuela A., Nuño-Soriano A., Fuentes-Vacas M., Góngora R., Hernández Á.P., Fuentes M. (2024). Functional proteomics based on protein microarray technology for biomedical research.. Adv. Protein Chem. Struct. Biol..

[12] Lin C.C., Chen C.S. (2025). Bacterial proteome microarray technology in biomedical research.. Trends Biotechnol..

[13] Bodulev O.L., Sakharov I.Y. (2022). Modern methods for assessment of microRNAs.. Biochemistry.

[14] Green M.R., Sambrook J. (2022). Analysis of RNA by northern blotting.. Cold Spring Harb Protoc.

[15] Wu Y., Yu C.R. (2022). Protocol to detect RNAs from tissue sections in mice using Y-branched probe in situ hybridization.. STAR Protoc..

[16] Voss G., Ceder Y. (2023). Two-Tailed RT-qPCR for the Quantification of A-to-I-Edited microRNA Isoforms.. Curr. Protoc.

[17] Zendjabil M. (2024). Preanalytical, analytical and postanalytical considerations in circulating microRNAs measurement.. Biochem. Med..

[18] Harshitha R., Arunraj D.R. (2021). Real-time quantitative PCR: A tool for absolute and relative quantification.. Biochem. Mol. Biol. Educ..

[19] Lin B., Jiang J., Jia J., Zhou X. (2022). Recent advances in exosomal miRNA biosensing for liquid biopsy.. Molecules.

[20] Boonbanjong P., Treerattrakoon K., Waiwinya W., Pitikultham P., Japrung D. (2022). Isothermal amplification technology for disease diagnosis.. Biosensors.

[21] Cheng Y., Dong L., Zhang J., Zhao Y., Li Z. (2018). Recent advances in microRNA detection.. Analyst.

[22] Li H., Cai Q., Wu D., Jie G., Zhou H. (2022). Fluorescence energy transfer biosensing platform based on hyperbranched rolling circle amplification and multi-site strand displacement for ultrasensitive detection of miRNA.. Anal. Chim. Acta.

[23] Gong Y., Zeng H., Gao S., He Y., Wang J., Wei J., Tang Q., Zhang K., Liao X. (2024). Application of CRISPR/Cas12a in miRNA-155 detection: A novel homogeneous electrochemiluminescence biosensor.. Anal. Chim. Acta.

[24] Wei J., Zhang J., Wang W., Zhou H., Ma H., Gong Y., Tang Q., Zhang K., Liao X. (2024). Precision miRNA profiling: Electrochemiluminescence powered by CRISPR-Cas13a and hybridization chain reaction.. Anal. Chim. Acta.

[25] Wu D., Liu X., Tang L., Li B., Zhao R., Feng T., Xiao X., Xie G., Feng W. (2024). Three-way junction-mediated three-letter coded SDA cascade CRISPR/Cas12a system for circRNA detection.. Chem. Eng. J..

[26] Ye J.W. (2020). Novel microRNA detection methods based on isothermal nucleic acid amplification technology.. Master's thesis, Zhejiang University.

[27] Niu C., Liu J., Xing X., Zhang C. (2023). Exploring the trans-cleavage activity with rolling circle amplification for fast detection of miRNA.. Biodes Res..

[28] Lee Y.J., Jeong J.Y., Do J.Y., Hong C.A. (2023). Rapid and ultrasensitive miRNA detection by combining endonuclease reactions in a rolling circle amplification (RCA)–based hairpin DNA fluorescent assay.. Anal. Bioanal. Chem..

[29] Zhang B., Li M., Wei Y., Wang J., Wang Y., Shi P., Tang H., Song Z. (2022). Detection of target DNA with a visual CRISPR-associated hyperbranched rolling circle amplification technique.. Anal. Biochem..

[30] Xie J., Chen J., Zhang Y., Li C., Liu P., Duan W.J., Chen J.X., Chen J., Dai Z., Li M. (2024). A dual-signal amplification strategy based on rolling circle amplification and APE1-assisted amplification for highly sensitive and specific miRNA analysis for early diagnosis of alzheimer’s disease.. Talanta.

[31] Song J., Ju Y., Kim S., Kim H., Park H.G. (2022). Palindromic hyperbranched rolling circle amplification enabling ultrasensitive microRNA detection.. Chem. Commun..

[32] Shao M., Wang S., Wang B., Guo L., Cui Y., Wei Y. (2024). Sensitive, low-background-signal mirna analysis via the self-priming-initiated color reaction loaded on a rolling circle amplification product.. ACS Omega.

[33] Zhou S., Sun H., Dong J., Lu P., Deng L., Liu Y., Yang M., Huo D., Hou C. (2023). Highly sensitive and facile microRNA detection based on target triggered exponential rolling-circle amplification coupling with CRISPR/Cas12a.. Anal. Chim. Acta.

[34] Zhang B., Li S., Guan Y., Yuan Y. (2021). Accurate detection of target MicroRNA in mixed species of high sequence homology using target-protection rolling circle amplification.. ACS Omega.

[35] Dong M., Tang Z., Hicks S., Guan W. (2022). Rolling circle amplification-coupled glass nanopore counting of mild traumatic brain injury-related salivary miRNAs.. Anal. Chem..

[36] Yang H., Niu H., Zhao C., Zhang S., Sun S., Shi P. (2024). A rolling circle amplification-based DNAzyme walker against intracellular degradation for imaging tumor cells’ microRNA.. Chem. Commun..

[37] Notomi T., Okayama H., Masubuchi H., Yonekawa T., Watanabe K., Amino N., Hase T. (2000). Loop-mediated isothermal amplification of DNA.. Nucleic Acids Res..

[38] Atçeken N., Yigci D., Ozdalgic B., Tasoglu S. (2022). CRISPR-cas-integrated LAMP.. Biosensors.

[39] Benke E.H., Boegner D.J., Bogusch A.M., White I.M. (2025). Sample-to-answer detection of miRNA from whole blood using thermally responsive alkane partitions.. Biosens. Bioelectron..

[40] Yang N., Zhang H., Han X., Liu Z., Lu Y. (2024). Advancements and applications of loop-mediated isothermal amplification technology: A comprehensive overview.. Front. Microbiol..

[41] Nam D., Kim S., Kim J.H., Lee S., Kim D., Son J., Kim D., Cha B.S., Lee E.S., Park K.S. (2023). Low-temperature loop-mediated isothermal amplification operating at physiological temperature.. Biosensors.

[42] Park J.W. (2022). Principles and applications of loop-mediated isothermal amplification to point-of-care tests.. Biosensors.

[43] Abdullah AL-maskri A.A., Ye J., Talap J., Hu H., Sun L., Yu L., Cai S., Zeng S. (2020). Reverse transcription-based loop-mediated isothermal amplification strategy for real-time miRNA detection with phosphorothioated probes.. Anal. Chim. Acta.

[44] Wu S., Abdullah Al-Maskri A.A., Li Q., Liu J., Cai S. (2023). A novel miRNA detection method using loop-mediated isothermal amplification.. Int. J. Anal. Chem..

[45] Hasegawa T., Hapsari D., Iwahashi H. (2021). RNase H-dependent amplification improves the accuracy of rolling circle amplification combined with loop-mediated isothermal amplification (RCA-LAMP).. PeerJ.

[46] Liu X., Peng H., Ye X., Zhang X., Xu G., Zhao X. (2025). A versatile colorimetric diagnostic platform based on primer exchange reaction cascades driven loop-mediated isothermal amplification.. Anal. Chem..

[47] Cao Z., Jiang X., Xiao G., Xu M., Liu H., Cai S. (2021). Rolling circle and loop mediated isothermal amplification strategy for ultrasensitive miRNA detection.. Separations.

[48] Zhang M., Wang H., Wang H., Wang F., Li Z. (2021). CRISPR/Cas12a-assisted ligation-initiated loop-mediated isothermal amplification (CAL-LAMP) for highly specific detection of microRNAs.. Anal. Chem..

[49] Su J., Wang M., Lin P., Huang Z., Li G., Chen X., Yan H., Zhou L. (2023). Trigger-activated autonomous DNA machine for amplified liver cancer biomarker microRNA21 imaging.. Anal. Sci..

[50] Huang G., Li C., Wu R., Xue G., Song Q., Lan L., Xue C., Xu L., Shen Z. (2024). Self-assembly of protein-DNA hybrids dedicated to an accelerated and self-primed strand displacement amplification for reinforced serum microRNA probing.. Anal. Chim. Acta.

[51] Bai J., Shu E., Zhang T., Fan H. (2023). Identification, cleavage, and amplification (ICA): A versatile strategy for highly sensitive detection of miRNA.. Anal. Chim. Acta.

[52] Jiang H., Li Y., Lv X., Deng Y., Li X. (2023). Recent advances in cascade isothermal amplification techniques for ultra-sensitive nucleic acid detection.. Talanta.

[53] Shi C., Liu Q., Ma C., Zhong W. (2014). Exponential strand-displacement amplification for detection of microRNAs.. Anal. Chem..

[54] Chi Z., Wu Y., Chen L., Yang H., Khan M.R., Busquets R., Huang N., Lin X., Deng R., Yang W., Huang J. (2022). CRISPR-Cas14a-integrated strand displacement amplification for rapid and isothermal detection of cholangiocarcinoma associated circulating microRNAs.. Anal. Chim. Acta.

[55] Xie Q., Chen J., Zhang J., Chu Z., Zhang F., Wang Q. (2024). Quantification of multiple microRNAs by microchip electrophoresis assisted by strand displacement amplification.. J. Chromatogr. A.

[56] Xu Y., Chen B., He M., Yuan G., Hu B. (2025). Dual-amplification single-particle ICP-MS strategy based on strand displacement amplification–CRISPR/Cas12a amplification for homogeneous detection of miRNA.. Anal. Chem..

[57] Zhou B., Fan T., Chen H., Huang Y., Lai X., Feng S., Ding Z., Chen Y., Jiang Y. (2025). Development of a multi-miRNA detection platform for enhanced diagnostics of colorectal cancer.. Biosens. Bioelectron..

[58] Wei B., Huang B., Zhao X. (2023). An overview of biochemical technologies for the cancer biomarker miR-21 detection.. Anal. Sci..

[59] Yan Y., Zhao H., Fang Y., Ma C., Chen J. (2022). Label-free miRNA-21 analysis based on strand displacement and terminal deoxynucleotidyl transferase-assisted amplification strategy.. Biosensors.

[60] Li J.H., Liu J.L., Zhang X.L., Zhu X.C., Yuan R., Chai Y.Q. (2023). Ultrasensitive electrochemiluminescence biosensor based on 2D Co 3 O 4 nanosheets as a coreaction accelerator and highly ordered rolling DNA nanomachine as a signal amplifier for the detection of MicroRNA.. Anal. Chem..

[61] Zada S., Lu H., Dai W., Tang S., Khan S., Yang F., Qiao Y., Fu P., Dong H., Zhang X. (2022). Multiple amplified microRNAs monitoring in living cells based on fluorescence quenching of Mo2B and hybridization chain reaction.. Biosens. Bioelectron..

[62] Jiang Z., Liu Z. (2024). Target recycle initiated entropy driven assembly strategy for sensitive, enzyme-free, and portable miRNA detection.. Anal. Biochem..

[63] Fan C., Xie L., Zhao F., Wang J., Lin X., Chen X. (2024). Novel fluorescence nano-orbital biosensor for highly sensitive microRNA detection.. Anal. Chim. Acta.

[64] Chen X., Huang C., Nie F., Hu M. (2023). Enzyme-free and sensitive method for single-stranded nucleic acid detection based on CHA and HCR.. Anal. Methods.

[65] Oishi M., Juji S. (2021). Acceleration of DNA hybridization chain reactions on 3D nanointerfaces of magnetic particles and their direct application in the enzyme-free amplified detection of microRNA.. ACS Appl. Mater. Interfaces.

[66] Ma X., Liu H., Tao S. (2023). A simple, sensitive and label-free method for miRNA analysis in gastric cancer via catalytic hairpin assembly assisted programming of split-G-quadruplexes.. Anal. Methods.

[67] Xie T., Liu Y., Xie J., Luo Y., Mao K., Huang C., Li Y., Zhen S. (2022). Catalyzed hairpin assembly-assisted DNA dendrimer enhanced fluorescence anisotropy for MicroRNA detection.. Chemosensors.

[68] Dong Z., Fan W., Ren W., Liu C. (2024). Bead-enriched catalyzed hairpin assembly for the flow cytometric detection of microRNA via FRET signal readout.. Sensors &. Diagnostics.

[69] Tian Z., Zhou C., Zhang C., Wu M., Duan Y., Li Y. (2022). Recent advances of catalytic hairpin assembly and its application in bioimaging and biomedicine.. J. Mater. Chem. B Mater. Biol. Med..

[70] Yang Y., Li J., Xiang S., Wang F., Yang H., Cai R., Tan W. (2024). PdPt@SnS 2 nanosheets for a novel ultrasensitive electrochemiluminescence biosensor for miRNA-21 assay.. Anal. Chem..

[71] Li J., Li H.W. (2023). Ultrasensitive miRNA detection by AuNP‐based 3D DNA walker and Catalytic Hairpin Assembly (CHA) cascade amplification for early cancer diagnosis.. Chem. Asian J..

[72] Zhang C., Zhao X., Chen X., Lin X., Huang Z., Hu J., Liu R., Lv Y. (2024). CRISPR/Cas12a assay for amol level microRNA by combining enzyme-free amplification and single particle analysis.. Chem. Commun..

[73] Gong K., Wu Q., Wang H., He S., Shanga J., Wang F. (2020). Autocatalytic DNAzyme assembly for amplified intracellular imaging.. Chem. Commun..

[74] Zeng Z., Zhou R., Sun R., Zhang X., Cheng Z., Chen C., Zhu Q. (2021). Nonlinear hybridization chain reaction-based functional DNA nanostructure assembly for biosensing, bioimaging applications.. Biosens. Bioelectron..

[75] Lu Y., Xu J., Liu S., Tian B., Long F. (2025). A facile optical fiber-embedded microfluidic biochip for rapid and sensitive detection of microRNA-let-7a in serum.. Mikrochim. Acta.

[76] Zhang Y., Liu L., Li C.M., Huang C.Z., Huang J. (2025). Highly sensitive detection of Let-7a in different tumor cells based on a dual cascade signal amplification strategy.. Anal. Chem..

[77] Tang Q., Liao Q., Huang X., Huang H., Tang Q., Zhang K., Liao X. (2025). A highly sensitive MiRNA detection sensor powered by CRISPR/Cas13a and entropy-driven amplification.. Bioelectrochemistry.

[78] Zhu Y., Zheng X., Zhou S., Xiao W., Sun X., Zhou J., Qian F., Zhang T., Sheng Y., Hu J. (2023). A dual amplification-based CRISPR/Cas12a biosensor for sensitive detection of miRNA in prostate cancer.. Anal. Chim. Acta.

[79] Cai R., Wu K., Chen H., Chen X., Zhang Y., Wang X., Zhou N. (2023). Nanosensor based on the dual-entropy-driven modulation strategy for intracellular detection of MicroRNA.. Anal. Chem..

[80] Huang Q., Wang K., Wang Y. (2024). Highly sensitive miRNA-21 detection with enzyme-free cascade amplification biosensor.. Talanta.

[81] Kim H.Y., Song J., Park H.G. (2021). Ultrasensitive isothermal method to detect microRNA based on target-induced chain amplification reaction.. Biosens. Bioelectron..

[82] Ou X., Li K., Liu M., Song J., Zuo Z., Guo Y. (2024). EXPAR for biosensing: Recent developments and applications.. Analyst.

[83] Jia H., Li Z., Liu C., Cheng Y. (2010). Ultrasensitive detection of microRNAs by exponential isothermal amplification.. Angew. Chem. Int. Ed..

[84] Zhang W., Song Y., Deng D., Liu M., Chen H., Zhang W., Lei H., Li Z., Luo L. (2025). Exponential rolling circle amplification-hybridization chain reaction (EXRCA-HCR) for AgNPs@gel-enhanced fluorescence ultrasensitive detection of miRNA-21.. Anal. Chim. Acta.

[85] Yuan C., Fang J., Luo X., Zhang Y., Huang G., Zeng X., Xia K., Li M., Chen X., Yang X., de la Chapelle M.L., Fu W. (2022). One-step isothermal amplification strategy for microRNA specific and ultrasensitive detection based on nicking-assisted entropy-driven DNA circuit triggered exponential amplification reaction.. Anal. Chim. Acta.

[86] Qian J., Zhang Q., Liu M., Wang Y., Lu M. (2022). A portable system for isothermal amplification and detection of exosomal microRNAs.. Biosens. Bioelectron..

[87] Yao C., Liu X., Lu X., Wang L., Jia J., Li Z. (2024). Smartphone-based fluorescent profiling of quaternary MicroRNAs in urine for rapid diagnosis of urological cancers using a multiplexed isothermal exponential amplification reaction.. Anal. Chem..

[88] Pan H.Y., Dong J.H., Shen X.Y., Zhao L.L., Liu Y., Chen Y.N., Huang Z.Q., Zhou Y.L., Zhang X.X. (2025). The fabrication of 1,2-dicarbonyl compound-caging isothermal exponential amplification strategy and its application in the highly sensitive detection of tumor exosomal miRNA.. Analyst.

[89] Peng J., Hang X., Wang H., Zhao K., Wang H., Yu H., Wang L. (2024). Low background self-primer EXPAR coupled with colorimetric and lateral flow assay for rapid and sensitive point-of-care detection of miRNA.. Sens. Actuators B Chem..

[90] Copeland C.E., Kwon Y. (2023). Suitability evaluation of toehold switch and EXPAR for cell-free MicroRNA biosensor development.. Biotechnol. Notes.

[91] Emaus M.N., Anderson J.L. (2021). Magnetic ionic liquids as microRNA extraction solvents and additives for the exponential amplification reaction.. Anal. Chim. Acta.

[92] Niu C., Liu J., Xing X., Zhang C. (2023). CRISPR-Cas12a-assisted elimination of the non-specific signal from non-specific amplification in the exponential amplification reaction.. Anal. Chim. Acta.

[93] Ang Y.S., Yung L.L. (2024). Robust sequence design space for the isothermal exponential amplification of short oligonucleotides.. Small.

[94] Yan X., Wang Q., Yang P., Liu Y., Liu B., Wang T., Qiu D., Wei S., Chen D., Zhou J., Liu C., Zhang X. (2025). Amplification bias-free sequence-generic exponential amplification reaction.. Anal. Chem..

[95] Reid M.S., Paliwoda R.E., Zhang H., Le X.C. (2018). Reduction of background generated from template-template hybridizations in the exponential amplification reaction.. Anal. Chem..

[96] Song Z., Hu K., Rao J., Cheng B., Xu L., An R., Liang X. (2023). Unexpected mechanism and inhibition effect for nonspecific amplification involving dynamic binding of primers with background DNA.. Anal. Chem..

[97] Yu H., Weng Z., Zhou X. (2023). A hairpin probe-mediated exponential amplification reaction for highly sensitive and specific detection of microRNAs.. Chem. Commun..

[98] Reid M.S., Le X.C., Zhang H. (2018). Exponential isothermal amplification of nucleic acids and assays for proteins, cells, small molecules, and enzyme activities: An EXPAR example.. Angew. Chem. Int. Ed..

[99] Chen J., Zhou X., Ma Y., Lin X., Dai Z., Zou X. (2016). Asymmetric exponential amplification reaction on a toehold/biotin featured template: An ultrasensitive and specific strategy for isothermal microRNAs analysis.. Nucleic Acids Res..

[100] Guo H., Chen J., Feng Y., Dai Z. (2024). A simple and robust exponential amplification reaction (EXPAR)-based hairpin template (exp-Hairpin) for highly specific, sensitive, and universal MicroRNA detection.. Anal. Chem..

[101] Wang Z.Y., Sun M.H., Zhang Q., Li P.F., Wang K., Li X.M. (2023). Advances in point-of-care testing of microRNAs based on portable instruments and visual detection.. Biosensors.

[102] Mao J., Tang S., Liang S., Pan W., Kang Y., Cheng J., Yu D., Chen J., Lou J., Zhao H., Zhou J. (2021). A new self-passivating template with the phosphorothioate strategy to effectively improve the detection limit and applicability of exponential amplification reaction.. Anal. Methods.

[103] Tachibana A., Saito S., Fujiyama Y., Tanabe T. (2020). LidNA, a miRNA inhibitor constructed with unmodified DNA, requires an xxxA insertion sequence in miRNA binding site for its potent inhibitory activity.. FEBS Lett..

[104] Zhu C., Zhu L., Tan D., Qiu X., Liu C., Xie S., Zhu L. (2019). Avenues toward microRNA detection in vitro: A review of technical advances and challenges.. Comput. Struct. Biotechnol. J..

[105] Wang J., Zou B., Rui J., Song Q., Kajiyama T., Kambara H., Zhou G. (2015). Exponential amplification of DNA with very low background using graphene oxide and single-stranded binding protein to suppress non-specific amplification.. Mikrochim. Acta.

[106] Trinh M.P., Carballo J.G., Adkins G.B., Guo K., Zhong W. (2020). Physical and chemical template-blocking strategies in the exponential amplification reaction of circulating microRNAs.. Anal. Bioanal. Chem..

[107] Deng Y.H. (2023). Improved exponential isothermal amplification reactions: Methodological refinements and practical applications.

[108] Lo C.Y., Gao Y. (2022). DNA polymerase-parental DNA interaction is essential for helicase-polymerase coupling during bacteriophage T7 DNA replication.. Int. J. Mol. Sci..

[109] Garafutdinov R.R., Kupova O.Y., Sakhabutdinova A.R. (2024). Influence of nucleotide context on non-specific amplification of DNA with Bst exo– DNA polymerase.. Biochemistry.

[110] Garafutdinov R.R., Gilvanov A.R., Kupova O.Y., Sakhabutdinova A.R. (2020). Effect of metal ions on isothermal amplification with Bst exo- DNA polymerase.. Int. J. Biol. Macromol..

[111] Arianna G.A., Korzhnev D.M. (2024). Protein assemblies in translesion synthesis.. Genes.

[112] Armitage J.D., Bolitho E.M., Moodley Y.P., Tan D.B.A. (2020). A simplified protocol for profiling heparin-contaminated circulating miRNAs: By microfluidic array.. Mol. Biol. Rep..

[113] Plieskatt J.L., Feng Y., Rinaldi G., Mulvenna J.P., Bethony J.M., Brindley P.J. (2014). Circumventing qPCR inhibition to amplify miRNAs in plasma.. Biomark. Res..

[114] Sriram H., Deshpande N., Tembhare P.R., Hasan S., Gujral S., Subramanian P.G., Patkar N.V., Rajpal S., Chatterjee G., Ghogale S., Tyagi P., Kedia S., Khanka T. (2021). Improved protocol for plasma microRNA extraction and comparison of commercial kits.. Biochem. Med..

[115] Giallombardo M., Chacártegui Borrás J., Castiglia M., Van Der Steen N., Mertens I., Pauwels P., Peeters M., Rolfo C. (2016). Exosomal miRNA analysis in non-small cell lung cancer (NSCLC) patients’ plasma through qPCR: A feasible liquid biopsy tool.. J. Vis. Exp..

[116] Wilkinson A.F., Barra M.J., Novak E.N., Bond M., Richards-Kortum R. (2024). Point-of-care isothermal nucleic acid amplification tests: Progress and bottlenecks for extraction-free sample collection and preparation.. Expert Rev. Mol. Diagn..

[117] Schönberg J., Borlak J. (2021). Reliable miRNA biomarker quantification in clinical practice – are we there yet?. Anal. Biochem..

[118] Marabita F., de Candia P., Torri A., Tegnér J., Abrignani S., Rossi R.L. (2016). Normalization of circulating microRNA expression data obtained by quantitative real-time RT-PCR.. Brief. Bioinform..

[119] Bustin S.A., Benes V., Garson J.A., Hellemans J., Huggett J., Kubista M., Mueller R., Nolan T., Pfaffl M.W., Shipley G.L., Vandesompele J., Wittwer C.T. (2009). The MIQE guidelines: Minimum information for publication of quantitative real-time PCR experiments.. Clin. Chem..

[120] Ishii Y., Aoki K., Oda M. (2022). A study of quality assessment in SARS-CoV-2 pathogen nucleic acid amplification tests performance; from the results of external quality assessment survey of clinical laboratories in the Tokyo Metropolitan Government external quality assessment program in 2020.. J. Infect. Chemother..

[121] Androvic P., Romanyuk N., Urdzikova-Machova L., Rohlova E., Kubista M., Valihrach L. (2019). Two-tailed RT-qPCR panel for quality control of circulating microRNA studies.. Sci. Rep..

